# Anesthesiological Considerations in Shoulder Surgery

**Published:** 2012-04-30

**Authors:** M. Lanna, A. Pastore, C. Policastro, C. Iacovazzo

**Affiliations:** 1 Assistant in training at Department of Anesthesiology and Intensive Care AOU“Federico II” Naples.; 2 Department of Anesthesiology and Intensive Care AOU “ Federico II” Naples.

**Keywords:** Shoulder surgery, brachial plexus, interscalene block, postoperative analgesia

## Abstract

In 1970, Winnie proposed the brachial plexus block as an alternative and effective anaesthesia technique for shoulder surgery. From that date, several techniques have been developed to approach the brachial plexus: the use of a nerve stimulator and, more recently, the ultrasound guided nerve blockade have made the procedure easier and more effective; the availability of the new drugs demonstrates some major advantages due to the application of peripheral blocks. Nowadays the attention has been focused on postoperative pain control: although many techniques have been proposed, the application of a continuous infusion of local anaesthetics through an interscalene catheter seems the best available technique to achieve pain relief after shoulder surgery. Advantages ad disadvantages of regional anaesthesia and adverse events associated with interscalene brachial plexus blockade are reviewed.

## I. INTRODUCTION

Until 70’s almost all the procedures for shoulder surgery were performed under general balanced anaesthesia. In 1970 Winnie first proposed the interscalene brachial plexus block as an alternative and effective anesthesiological technique for shoulder surgery (1). The author demonstrated that the interscalene space is a continuous, fascial-enclosed sheat containing both the brachial plexus and the cervical plexus and because the shoulder is innervated by nerves from both plexuses, a single injection into this space following anatomic landmarks would provide a satisfactory anaesthesia for shoulder. From that date, several techniques have been developed to approach the brachial plexus, but only in the last ten years there has been renewed interest in regional anaesthesia. This is due to the availability of the new drugs (minor toxicity, long action) and new materials and to the results of studies demonstrating some major advantages linked to the application of peripheral blocks rather then general anaesthesia, particularly in outdoor patients. The use of a nerve stimulator and, more recently, the ultrasound guided nerve blockade have made the procedure easier and more effective, with high percentage of success and less complications. In the last few years, focused attention has been paid to postoperative pain control. Interscalene nerve blockade provides a better and prolonged analgesia, thus allowing early rehabilitation and good surgical outcome. Although many techniques have been proposed, the application of a continuous infusion of local anaesthetics through an interscalene catheter looks like the best technique available to achieve pain relief after shoulder surgery.

## II. SELECTION OF ANESTHETIC TECNIQUE

Shoulder surgery can be performed under regional or general anaesthesia. Interscalene brachial plexus block has been used for a variety of procedures about the shoulder, including instability repairs, proximal humeral prosthetic replacements, total shoulder arthroplasties, anterior acromioplasties, rotator cuff repairs, and operative treatment of humeral fractures, for both arthroscopic or open approach. These blocks have resulted in good surgical anaesthesia, a minimum of complications, and a high degree of patient acceptance (2).

Regional anaesthesia has been credited with having several advantages over general anaesthesia for shoulder surgery. These include excellent muscle relaxation, less blood loss, shorter hospital stay, reduced postoperative analgesia requirements, and avoidance of the risks and side effects of general anaesthesia, especially in patients with underlying medical conditions in whom general anaesthesia may place the patient at significant risk (3). Vantages and disadvantages of regional anaesthesia are shown in table 1.

One criticism directed against the use of regional anaesthesia is the argument that it adds significantly more time to the procedure than general anaesthesia, this influencing also the cost-effectiveness of the technique. Brown et al. demonstrate that there is no difference on the average time from the start of administration of anaesthetic to the incision in the general anaesthesia versus interscalene block. Positioning and draping the patient while the block is setting helps to minimize the waiting time. At the end of the procedure the patient is wide awake and can leave the operating room immediately. This is not always the case of general anaesthesia, in which delays can occur due to slow awakening patient, slow reversal of muscle relaxation, and the need to extubate the patient. A further improvement is the insertion of the block in a regional anaesthesia block room before the patient enters the operating room. This is performed while the operating room is being cleaned and set up, reducing the turnover time (2).

During the last few years, ultrasonographic guidance may reduce procedure times for regional techniques and thereby influence cost-effectiveness. Other aspects, such as decreased time to discharge or reduced demand for postoperative analgesic, may increase cost-effectiveness of regional techniques. Gonano et al. demonstrate that the cost reduction of ultrasonographic-guided ISB during shoulder surgery is significant when compared with general anaesthesia, even if they didn’t consider the investment associated with ultrasound equipment and training (4).

Other concerns about the routine use of interscalene blocks for shoulder surgery are due to the variable rate of successful block placement, from 84% to 98% according to case series (5, 6), and the possibility of major complications, including inadvertent spinal/epidural anaesthesia, seizure and cardiac arrest (7). Although the more serious complications of interscalene blocks are rare, they may be life-threatening. For this reason patient should be fully monitored during the insertion of the block, and resuscitation equipment should be immediately available. Technical factors associated with block failure include inadequate volume of local anaesthetic agent, incorrect needle placement, inadequate explanation of possibly frightening but harmless side effects such as hoarseness and shortness of breath, and lack of adequate sedation for the more anxious patients (2). Adverse events that may occur with an interscalene brachial plexus blockade are shown in table 2.

Few data exist on the combined use of general anaesthesia and intraoperative or postoperative interscalene blocks. In principle, combined anaesthetics will lead to additive complication risk. Arcas et al. reported a case of quadriparesis after combined interscalene block and general anaesthesia (8) probably due to the central migration of the anaestetics. The indication for combined anaesthesia include difficult airway management when profound sedation is needed, longer procedures especially when the lateral decubitus is adopted, and the inclusion of an iliac crest bone graft as a part of surgical procedure (3). The interscalene block should only be performed before the induction of general anaesthesia, as it is associated with potentially serious complications. Benumof described four cases in which interscalene block performed after a general anaesthesia led to a total spinal anaesthesia and to extensive permanent cervical spinal cord damage (9).

## III. INTERSCALENE BLOCK

The shoulder area is innervated by nerves of both cervical and brachial plexuses.

The shoulder joint is supplied by the anterior primary divisions of cervical nerve roots C5-C6 (with a small contribution of C7), while the cutaneous innervation of the shoulder is predominantly derived from C4-C3 (superficial cervical plexus). An interscalene block will consistentely block C4 and C3 as well as C5, C6, and C7. Cervical nerve roots C8 and T1 are blocked approximately 40 to 60 % of patients. The commonly used superior and deltopectoral surgical approaches are within the dermatomes anesthetized by an ISB. The lower anterior aspect of the shoulder, and the dorsal aspect as well, are innervated by thoracic nerve roots T2 and T3. These areas can be blocked combining an ISB with a subcutaneous infiltration of local anesthetic to cover a larger surgical incision (3). The acromioclavicular joint is largely supplied by the soprascapular nerve, which also provides some innervations to the capsule and glenohumeral joint. The inferior aspect of the capsule and glenohumeral joint are supplied by the axillary nerve, with a small variable contribution from the muscolocutaneous and subscapular nerves. To summarize, it is mandatory to block supraclavicular, suprascapular and axillary nerves for the arthroscopic surgery. For open shoulder surgery, knowledge of the surgical approach is useful because the surgical incision may also involve other territories (10).

The interscalene approach to the brachial plexus is best suited to surgery of the shoulder where a block of the lower cervical plexus is also desirable. Use a nerve stimulator or ultrasonographic guidance are possible techniques to place the local anesthetic solution accurately. Alternatively, percutaneous electrode guidance uses a transcutaneous stimulating probe with the nerve first located by eliciting the desired motor response at a current of 5 mA at an increased pulse width of 1 ms. The needle insertion point can be then mapped on the skin and the block needle introduced (11).

*Ultrasound-guided interscalene block* - When compared with traditional nerve stimulation technique, ultrasound guidance for interscalene block significantly reduces number of needle passes, procedure-related pain, required local anesthetic volume, and postoperative pain. Furthermore, ultrasound-guided blocks can be taught and learned more easily then traditional electrical stimulation techniques (12, 13).

McNaught et al. demonstrate that the ultrasound guidance reduce the minimum anesthetic volume (MEAV) 50 of ropivacaine 0,5% to approximately 5 ml, with fewer respiratory complications and no change in postoperative analgesia for the first 24 hours compared with the standard volume (20 ml) technique (14).

One systematic review has noted that the interscalene block has the highest incidence of permanent neurological complications of all peripheral nerve blocks. Part of this risk might be due to unrecognized intraneural injection. Unintended subepineural injection is probably common when a nerve stimulation alone is used to guide peripheral nerve blockade. Visualization of peripheral nerves with ultrasound has revealed that electrical stimulation of a nerve frequently does not occur, even when the needle tip is in direct contact with the external surface of the nerve (15). Ultrasound guidance may help to readily note intraneural injection in some patients, but in others it can be difficult to identify the boundaries of the nerves between the hypoechoic structures aligned in the interscalenic groove.

Furthermore, the neurostimulation technique for a plexus localisation specific for interscalene catheter placement has been shown to be associated with a false negative motor response rate of over 50%, and this is higher then that reported for single-injection techniques (16) and for ultrasound guided procedure. The latter, facilitating catheter positioning adjacent to the most appropriate elements of brachial plexus (C5-6 roots/superior trunk), has been recently shown to reduce local anesthetic and oral analgesic consumption in the postoperative period (17).

*Continuous interscalene block -* Continuous interscalene block was first described in 1987, using an approach similar to that described by Winnie for interscalene block; however, it was associated with failure rates as high as 25%. In 1997, the description of a new approach by Meier et al. resulted in a rise in its popularity and with it, increasing of its effectiveness. The essential modification was that the needle insertion point cranially at the level of C6 in the interscalene space, which theoretically promotes catheter threading in close proximity to the brachial plexus and enables the placement of sufficient catheter beneath the skin, thereby facilitating fixation (18). Early descriptions of the technique involved non-stimulating catheters, threated at least 5 cm beyond the needle tip. Electrical catheter stimulation was promoted as a way of precisely confirming catheter positioning and therefore of reducing failure rates (19). Finally, interscalene catheter placement, utilising a posterior approach analogous to that used for posterior approach in the single-shot procedure, has been shown to be an effective analgesic technique. More recently, the ultrasound-guided perineural catheter placement has gained popularity (see before).

## IV. DRUGS

Choice of local anesthetic agent is usually determined by the duration and magnitude of surgery. Lidocaine is appropriate for short procedure (e. g relocation of dislocated shoulder); however, for most surgery, a long-acting agent such as levobupivacaine or ropivacaine is more appropriate given the significant postoperative pain involved and the necessity of early rehabilitation for a good surgical outcome (20).

The average volume of drug needed for bolus application varies between 30 and 50 ml and has to be adapted first to the characteristic of the patient and second to the anesthetic technique, block alone or combined with general anaesthesia. The expected duration of block varies between 3 and 5 hours with mepivacaine 1 or 2 % and lidocaine 1,5 % and between 8 and 12 hours with bupivacaine 0,5 % and ropivacaine 0,5 % or 0,75 %. The duration of action is also proportional to the volume administered.

It is common practice to use a mixture of short and long-acting local anesthetic to reduce the onset and prolong the duration of the nerve block. Surprisingly, Gadsen et a. demonstrate that the addition of mepivacaine 1,5% to bupivacaine 0,5% results in a block onset similar to using bupivacaine alone, but the mean duration of blockade with such mixture was shorter then the block with bupivacaine 0,5% alone, according to patient interpretation (21).

Clonidine at a minimal dose of 0,5 microg/kg, but not opiods, has a prolonged duration of both anaesthesia and analgesia. The addition of epinephrine may prolong the duration of action of short-acting local anesthetic, but the risk of nerve ischemia must be kept in mind. The addition of clonidine or epinephrine increase the duration of motor block as well (10).

The most commonly used local anesthetic agents for infusion are levobupivacaine and ropivacaine, infused at low concentration to avoid prolonged motor block (e. i. Bupivaciane 0,15% or Ropivacaine 2% at 5 ml/h). An additional patient controlled component (2,5 ml to 4 ml bolus every 20–30 min) may be useful to increase efficacy (10,20), because of the dynamic nature of pain, which is moderate to severe at rest to very severe during movement. This strategy allows to rapidly reinforce the block shortly before and after the physical therapy session.

Borgeat et al. demonstrate that continued interscalene infusion with ropivacaine 2% compared to that with bupivacaine 0,15% was associated with better preservation of hand strength 24 hours and 48 hours after the beginning of the infusion as well as 6 hours after the infusion was stopped, with a significantly higher incidence of residual paraesthesias in the bupivacaine group (22).

The concentration of ropivacaine 0,2% is adequate for most patient but some authors suggest to increase up to 0,3 or 0,4% in young, athletic patient (10). Frederickson et al. demonstrate that a background infusion of ropivacaine of at least 4 ml/h is required for optimal analgesia, but equally important is the bolus dose, the optimal volume which appears to be at least 4 ml. There appears to be little benefit in administrating concentration of ropivacaine > 2% (23).

## V. POSTOPERATIVE ANALGESIA

Early and efficient rehabilitation on the first postoperative day is necessary for improving outcome after shoulder surgery and pain is the major factor which compromises early physical therapy. Therefore excellent postoperative analgesia is essential to provide a good functional recovery.

Shoulder surgery is associated with severe to very severe pain, particularly after surgery to the rotator cuff, and can be significant for 48 hours. Although it is commonly claimed that arthroscopic procedure can reduce early postoperative pain, these benefits are typically only seen after the first few days (24).

One of the characteristics of this pain is its dynamic component. From moderate at rest, it becomes most severe during mobilization. Up to 70 % of patients reported severe pain on movement after open major shoulder surgery, which is more than after gastrectomy and thoracotomy, requiring high doses of opioids for several days. The reason for this is that major joint operations entail massive nociceptive input form the richly innervated joint tissues that produce continuous deep somatic pain and bouts of severe reflex spasm of muscles supplied by the same and adjacent spinal cord segment supplying the site of surgery. Moreover, periarticular structures exhibit not only C afferents but also A-alpha and A-delta afferents, the latter being poorly blocked by opioids (25).

The use of opioid only is associated with different adverse effects, including nociception-induced central sensitization and secondary hyperalgesia. Both mechanism may be involved in the pathogenesis of persistent post-surgical pain, an entity that can occur following many shoulder procedures (26).

Thus, a multimodal approach and opioid-sparing techniques are required to achieve adequate postoperative analgesia. Analgesic options include (27):

### Conventional oral and parenteral analgesia (NSAIDs)

#### Patient-controlled analgesia

*Interscalenic analgesia -* (single-shot or continuous infusion or patient controlled bolus of local anesthetic- PCIA- patient controlled interscalene analgesia). The limited duration of the single-shot approach makes it suitable just for minor arthroscopic surgery, but it still a very useful technique, particularly when the expertise and logistics required for continuous interscalene analgesia are unavailable (28). An interscalene block with bupivacaine provides analgesia for about 15 hours, so rescue analgesia, usually a strong opioid, must be available when the block regresses (20).

Continuous interscalene block provides better analgesia, improves patient satisfaction and reduces opioid-related side effects (29). Of all the peripheral nerve block techniques, the interscalene approach is possibly the most suited to a continuous technique. This is because of the prolonged severe pain associated with shoulder surgery, the anatomical advantages that a single catheter can be used to block the shoulder joint, and the fact that any resulting motor block is generally well tolerated (27). The interscalene catheter is indicated in almost all open shoulder surgeries, the rotator cuff repair being the gold indication. According to the type of surgery performed, the catheter may be used for 3 to 5 days.

*Subacromial (bursal) or intra-articular infiltration of local anesthetic* - usually performed by the surgeon just before wound closure using a volume of 20–50 ml of local anesthetic (30). Intra articular injection with bupivacaine and morphine provides useful pain control and reduces morphine consumption in the first 24 h after major shoulder surgery. A standard epidural kit can be used to insert a catheter into the subacromial bursa at the end of the procedure, but the diluition of local anesthetic may be a significant factor in lowering the efficacy of the technique (31).

This technique was seen to be as a simple and effective alternative to scalene analgesia for only arthroscopic non-rotator cuff procedures; for open and/or rotator cuff procedures it appears to perform only marginally better that a placebo (27). More recently, concern has been raised over the possibility of iatrogenic chondrolysis associated with intrarticular local anesthetic, particularly with high doses of bupivacaine (32). Actually, this treatment modality can no longer be recommended.

*Suprascapular with or without axillary (circumflex) nerve blockade combined with local anaesthetic wound infiltration -* The suprascapular nerve provides sensory contributions to 70% of the joint capsule in addition to the subacromial bursa, the acromioclavicular joint and the coracoclavicular ligament; but it will not provide any cutaneous analgesia. The nerve is quickly blocked in the suprascapular fossa either with a landmark-only based technique (needle insertion site at 1 cm above the mid point of the scapular spine, at an angle perpendicular to the skin) or with the assistance of a nerve stimulator (contaction of supraspinatus and infraspinatus muscles) or ultrasound device. Concomitant blockade of the axillary nerve has been recently suggested by Price et al. to provide more complete shoulder joint analgesia (33, 34). This technique provides clinically significant improvement in pain control when compared to placebo, but inferior analgesia compared with interscalene block. The main advantages is the avoidance of motor block to those parts of the upper limb innervated by the more inferior roots of the brachial plexus (c8-t1), thus eliminating the risk of phrenic nerve blockade and it can be performed when a interscalene block is contraindicated. Major disadvantages include the requirement for two separate needlings, incomplete blockade of all nerves innervating shoulder joint, and limited duration of action. Adverse events include nerve damage, intravascular injection and pneumothorax (35). It can be used alone to facilitate reduction of shoulder dislocation.

## VI. CONCLUSIONS

In the last ten years the peripheral approach to shoulder surgery has showed some major advantages to general anaesthesia. These benefits include the administration of lower concentrations of anaesthetic drugs, a lower incidence of postoperative nausea and vomiting, reduced hospital admission rates, reduced postoperative analgesic requirements. Indeed, the control of pain is necessary not only for the patient’s well-being, it also as a positive impact on the outcome of surgery. The goal is to provide the best conditions for the patient in term of peri- and post-operative pain control and for meeting the surgical orthopaedic requirements in terms of favourable conditions for surgery and early and efficient rehabilitation. Interscalene block, especially when continued as an infusion, should be considered as the technique of choice for the large majority of patients having shoulder surgery. Despite advances in methods used to facilitate catheter placement, it remains a technically challenging procedure and is consequently under-utilised. In the future, the more extensive use of ultrasound approach will increase the number of continuous infusion brachial plexus block procedures, with less complications and high success rate.

## Figures and Tables

**Fig. 1:**
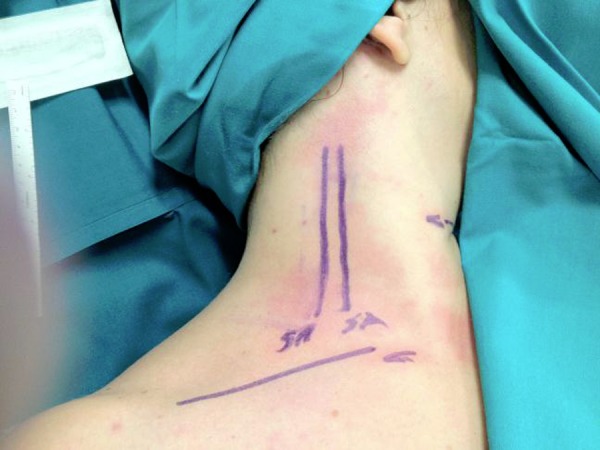
Anatomical landmarks: C (Clavicle) ; SA (Anterior Scalene Muscle); SM (Middle Scalene Muscle).

**Fig. 2:**
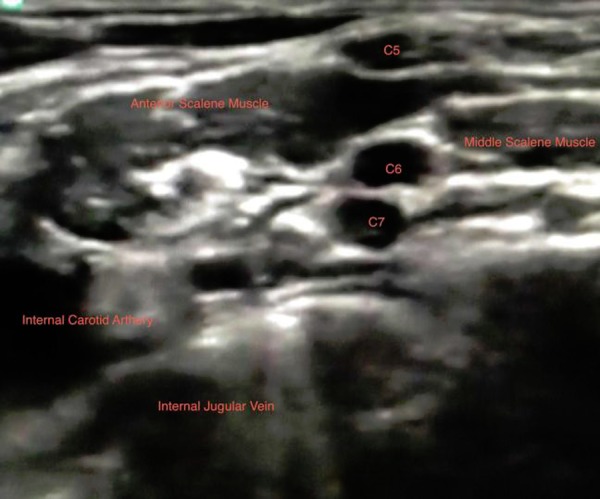
Sonographic anatomyof the brachial interscalenic plexus.

**TABLE. 1:** Advantages ad disadvantages of regional anaesthesia

**Advantages**	**Disadvantages**
Peri-operative pain control	Complication (including failure) particularly in unexperienced hands
Muscle relaxation limited to the operative limb	Addictional injections required
Less intraoperative bleeding	Control of a sedated patient’s airway
Improved operating room efficacy	Risk associated with an anesthetized limb
Decreased requirement of opiods	
Shorter recovery room and hospital stay	
Significant patient satisfaction	
Cost benefit	

**TABLE 2:** Adverse events associated with interscalene brachial plexus blockade.

**Symptoms/signs**	**Adverse event**
Seizure	Accidental vascular injection of local anesthetic
Cardiac toxicity	
Hoarseness	Recurrent laryngeal nerve blockade, cervical sympathetic blockade
Horner’s syndrome	Stellate ganglion blockade
Hemidiaphragmatic paresis	Phrenic nerve blockade
Quadriparesis, apnoea, brainstem and spinal cord toxicity	High epidural, total spinal, and subdural injection
Hypotensive-bradicardic events	Bezold-Jarish reflex (see below)
Others	Transient neuropathies, hematoma, pneumothorax, venous air embolism, subcutaneous emphysema, pneumomediastinum, etc
